# Neonatal Encephalopathy: Need for Recognition of Multiple Etiologies for Optimal Management

**DOI:** 10.3389/fped.2019.00142

**Published:** 2019-04-16

**Authors:** Saima Aslam, Tammy Strickland, Eleanor J. Molloy

**Affiliations:** ^1^Paediatrics, National Maternity Hospital, Dublin, Ireland; ^2^UCD School of Medicine & Medical Sciences, University College Dublin, Dublin, Ireland; ^3^Trinity College Translational Medicine Institute, Academic Paediatrics, Trinity College Dublin, National Children's Hospital, Dublin, Ireland; ^4^Paediatrics, Coombe Women's and Infant's University Hospital, Dublin, Ireland; ^5^Neonatology, Our Lady's Children's Hospital, Drimnagh, Ireland

**Keywords:** neonatal encephalopthy, etiology, antenatal, perinatal, targeted adjunctive therapies

## Abstract

Neonatal encephalopathy (NE) is associated with high mortality and morbidity. Factors predisposing to NE can be antenatal, perinatal, or a combination of both. Antenatal maternal factors, familial factors, genetic predisposition, hypoxic ischemic encephalopathy, infections, placental abnormalities, thrombophilia, coagulation defects, and metabolic disorders all have been implicated in the pathogenesis of NE. At present, therapeutic hypothermia is the only treatment available, regardless of etiology. Recognizing the etiology of NE involved can also guide investigations such as metabolic and sepsis workups to ensure optimal management. Understanding the etiology of NE may allow the development of targeted adjunctive therapies related to the underlying mechanism and develop preventative strategies.

## Introduction

**Figure 1:**
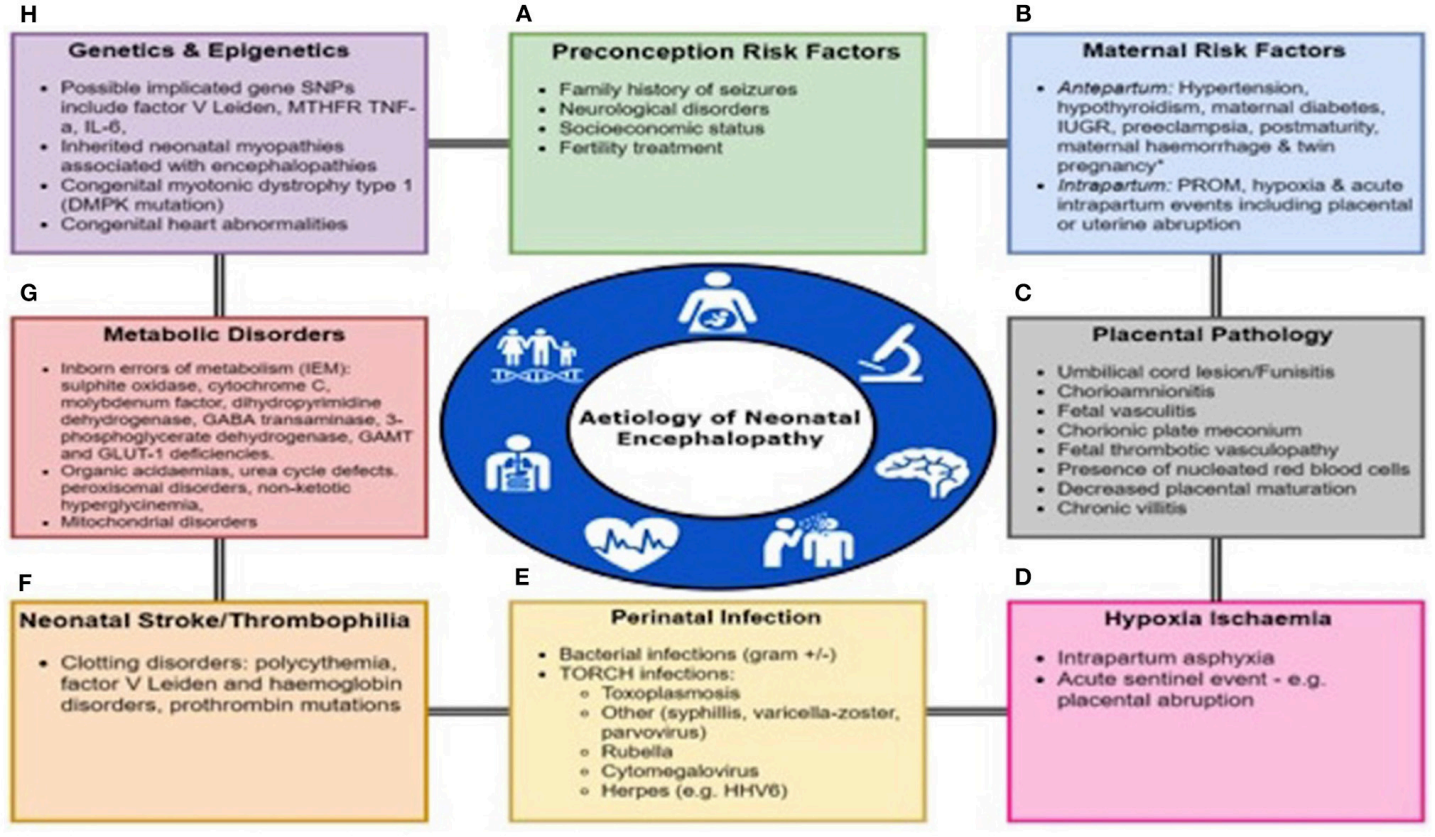
Multifactorial Etiology In Neonatal Encephalopathy. Many factors predispose to the onset of Neonatal Encephalopathy either alone or in a combination including **(A)** Preconception Risk Factors, **(B)** Maternal Antepartum/intrapartum comorbidities or events, **(C)** Placental pathology, **(D)** Hypoxia-ischaemia, **(E)** Perinatal infection, **(F)** Neonatal stroke or thrombophilia, **(G)** Metabolic disorders, and **(H)** Genetic and epigenetic abnormalities. PROM, prolonged rupture of membranes; IUGR, intrauterine growth restriction.

Neonatal encephalopathy (NE) is a complex disease of the newborn characterized by an altered level of consciousness, seizures, poor tone, an inability to initiate or maintain respiration (1) and is associated with multi organ dysfunction (2). The incidence of NE is estimated at 3 per 1,000 live births (3). NE can result from a wide variety of causes and is a clinical term that does not specify etiology. The only treatment available is therapeutic hypothermia (TH) with maximal benefit if initiated in first 6 h of life (4). There are multiple causes of NE such as hypoxic ischemic encephalopathy (HIE), perinatal infections, placental abnormalities, metabolic disorders, coagulopathies and neonatal vascular stroke (Figure 1). However, in more than half of the cases the cause of NE remains unidentified (5).

The use of the term NE vs. HIE is controversial. It has been proposed that in term and late preterm infants with no identifiable sentinel events the term NE should be used (6, 7). It is difficult to prove the presence of cerebral hypoxic ischemia apart from in animal models and particular cases of neonatal stroke. All current parameters including pH and seizures are non-specific (7). Some of the patterns of brain injury seen in NE patients can be produced in animal models by hypoxia-ischemia but this does not prove that all NE is due to HIE (7). The few published population-based studies have shown that antepartum and non-asphyxial risk factors are associated with NE (6). On the contrary some authors state that HIE is the cause of NE in 50 to 80% of cases based on clinical, EEG and MRI criteria (1). Therefore, for this review we have used the term NE and we aimed to look at the different etiologies associated with NE.

## Maternal Factors

Maternal hypertension (16%) was an important antenatal risk factors for NE in the Vermont Oxford Network Neonatal Encephalopathy Registry (*n* = 4,165). There was also a higher prevalence of clinical chorioamnionitis, prolonged rupture of membranes and maternal hypothyroidism. Although an acute asphyxial event was recorded in 15%, an association with inflammation was found in 24% (5).

A population-based study in Western Australia showed that socioeconomic status, family history of seizures, neurological problems and conception after fertility treatment were all independent preconception risk factors for NE (*n* = 164) (8). Maternal thyroid disease, bleeding during pregnancy, viral illness, preeclampsia, an abnormal placenta, intrauterine growth restriction and post maturity were other important antepartum risk factors (8). Identifiable antepartum risk factors for NE were found in 69% of cases, intrapartum risk factors in 5%, both ante- and intrapartum in 24% and no risk factor in 2%. In only 4% cases of NE intrapartum hypoxia alone was identified without any antepartum or preconception risk factors (9). Growth restriction in developed countries and twin pregnancy in the developing countries were found to be associated with NE. Maternal thyroid disease, antenatal care, infection and labor and delivery managements to be important modifiers in a study of 27 neonates with NE and 100 controls (3). Antepartum (74 vs. 18%), intrapartum (67 vs. 19%), and acute intrapartum events (33 vs. 2%) are more likely in infants with NE compared to controls. Therefore, in the neonatal clinical history a detailed maternal history is essential and may be a route to preventative strategies in the future.

In 45 neonates with NE 36% had sentinel event, 40% had chorioamnionitis and 11% had both. If sentinel events were taken out then maternal age > 35 years (RR, 2.5; 95% CI, 1.1–5.6) and urinary tract infection during pregnancy (RR, 2.6; 95% CI, 1.0–6.5) were potential antenatal risk factors for NE (10).

## Hypoxia-Ischaemia

Although the concept of hypoxia-ischaemia is clear cut in animal models it may not be the appropriate terminology to use in all cases of human neonatal NE. The “dose” and duration of hypoxia and the degree of ischaemia can be directly measured in an animal model. However, in human neonates this is unknown unless there is a sentinel event and subsequently surrogate markers of hypoxia are used. Badawi et al. showed that Intrapartum asphyxia was thought to be a contributing factor in 29% of cases of NE and a stand-alone factor in only 4% of cases (9). Intrapartum hypoxia-ischaemia was found to be a contributing factor in 30% of cases of NE in developed and 60% in developing countries (3). The American College of Obstetricians and the American Academy of Pediatrics defined a sentinel event in their document on Neonatal Encephalopathy and neurologic outcome (Obstet Gynecol 2014). The following are consistent with an acute peripartum or intrapartum event: a sentinel hypoxic or ischemic event occurring immediately before or during labor and delivery for example a ruptured uterus or severe abruptio placentae; fetal heart rate monitor patterns consistent with an acute peripartum or intrapartum event; timing and type of brain injury patterns based on imaging studies consistent with an etiology of an acute peripartum or intrapartum event and no evidence of other proximal or distal factors that could be contributing factors. In addition, the combination of low Apgars scores, acidaemia, abnormal cardiotocograph, MRI changes consistent with hypoxia-ischaemia and multiorgan dysfunction are all associated with a hypoxia-ischaemic etiology but equally can be altered by sepsis or other causes of NE. Asphyxial birth events and inflammation were only present in 12.6% of cases of Cerebral palsy (11). In neonates with NE (*n* = 405) the following were independently associated with NE (*p*-value = 0.001) including: only 1 antepartum (Gestational age> 41 weeks) and 7 intrapartum factors including prolonged rupture of membranes, abnormal cardiotocograph, thick meconium, sentinel events, shoulder dystocia, tight nuchal cord and failed vacuum (12). In a cohort of 26 neonates with NE sentinel events (aOR 74.9, 95% CI 11.9-infinity, *p* < 0.001) and category 3 fetal heart rate tracing (28.0% vs. 4.0%, *p* = 0.002) were strongly associated with NE (13). In a cohort of 45 neonates 36% had sentinel events and 11% had both sentinel events and chorioamnionitis (10).

Neuroimaging using Magnetic resonance imaging, conventional, diffusion, and spectroscopy from 24 h to 96 h of life gives a useful guide regarding the potential timing of a cerebral insult especially diffusion abnormalities. Abnormalities from a cerebral injury become most evident after 7 days using qualitative MRI and diffusion and spectroscopy are useful. Deep nuclear gray matter or watershed cortical injury evolve in a well-defined pattern of brain injury and are typical of hypoxic—ischaemic cerebral injury in the newborn. Genetic and metabolic causes may be pursued if a different pattern of brain injury or evolution of injury exists on MRI. Indications that peripartum hypoxia—ischemia was not causative of NE is suggested buy the following on MRI: porencephaly, focal arterial infarction, venous infarction, isolated intraparenchymal, or intraventricular hemorrhage, or atypical patterns of metabolic encephalopathies.

In NE associated with an acute sentinel event (HIE), the typical pattern of injury on MRI showed involvement of basal ganglia, thalami with associated posterior limb of the internal capsule and in severe cases brain stem involvement (14). In NE without a documented sentinel event, Basal ganglia or thalamic (BGT) involvement is also associated with white matter changes in 50% of cases (14). BGT with extensive white matter (WM) damage was found in 14%, BGT with mild to moderate WM in 56%, isolated thalamic injury in 5%, moderate WM damage only in 2% and mild WM or normal findings in 23% in 48 infants with HIE. The internal capsule was abnormal in 93% of patients with moderate to severe WM damage and was associated with death and CP in 86% of patients (15).

The pattern of brain injury may give indication of the possible cause for example, cortical and white matter injury (focal/multifocal) may be associated with placental insufficiency and chorioamnionitis. Early categorization of patients according to pattern of brain injury may lead to a different therapeutic approach as it has been shown in animal models that different levels of hypothermia protect against cortical or deep gray matter injury thus patient specific hypothermia protocols may be required for maximum benefit. Similarly, in animal models different modes of cooling that is systemic vs. selective result in different brain temperatures (16).

It is difficult to definitively establish the presence, duration and degree of hypoxia and ischemia in newborn and surrogate markers such as pH and seizures have been used (7). Therefore, biomarkers of multiorgan involvement may be helpful for diagnosis or to assess the severity of hypoxic ischemia and predict outcome in NE. Cardiac troponin is a marker of myocardial ischemia in neonates and children and is released from myocytes after injury to its membrane. Cardiac troponin I (cTnI) was significantly higher in neonates with HIE in first 48 h compared to controls (*p* < 0.0005) (17). Cardiac TnI was increased in neonates with higher grades of NE and those who required inotropes (18). A cut off value of <0.22 ng/ml for normothermic and <0.15 ng /ml for hypothermic infants with NE predicted normal neurodevelopmental outcome (19).

Hypoxia results in increase in erythropoiesis and release of immature nucleated red cells (nRBCs) into the circulation (20). Cord blood nRBC were significantly higher in asphyxiated neonates compared to controls and also were correlated with Apgar at 1 min, pH and development of NE (20). Nucleated red blood cells per 100 white cell count were higher in neonates with NE compared to controls, infants with moderate/severe vs. mild NE (*p* = 0.016) and in neonates with poor neurodevelopmental outcome (*p* = 0.03) (21). Therefore, a standardized measurement of clinical hypoxia exposure would be valuable using a combination of these markers and may assist in the timing of the insult and correlation with preclinical animal models.

## Infection

The risk of spastic cerebral palsy (CP) and quadriplegic CP was increased if there was a combination of infection and asphyxia (22). Interleukin (IL)-6 levels at 6 h is associated with abnormal neurological exam (23). NE, inotropic support, seizures without meningitis, need for intubation and lower 5 min apgar were more likely in neonates with CP if there was maternal infection compared to CP with no infection (22).

Maternal and early onset neonatal infections are a diagnostic challenge. There is debate whether occult infection not detectable by conventional culture methods may be associated with neurological injury in all age groups. Polymerase chain reaction (PCR) detected 11 cases of bacteraemia in encephalopathic neonates in Africa who had a negative blood culture (NE *n* = 201). PCR in combination with blood culture resulted in detection of bacterial products in 8.9% of cases of NE compared to 3.1% when blood culture used alone (24). In a meta-analysis Group B streptococcal (GBS) was associated with NE in 0.58% of cases and mortality was higher with GBS plus NE vs. NE alone (25). In a cohort of 45 neonates 40% had chorioamnionitis and 11% had both sentinel events and chorioamnionitis (10).

Therapeutic hypothermia delays the rise and peak response in c-reactive protein (CRP) as well-results in decrease in White cell count and neutrophil count compared to normothermic neonates (26). This effect of hypothermia on delaying the evolution of natural inflammatory markers should be considered when deciding antibiotic treatment and duration for neonates with NE.

Therefore, a complete sepsis evaluation is essential in all babies with NE. There is no standardized guideline about performing a lumbar puncture in all cases of NE and may be selected on a case-by-case basis. Infants with an elevated WCC and CRP or septic risk factors may be candidates. There are also technical and practical challenges for Lumbar Puncture in infants with NE who are rigid due to TH and also often initially have cerebral oedema on cranial ultrasound.

## Placental Abnormalities

Umbilical cord lesion, chorioamnionitis, fetal vasculitis, chorionic plate meconium and fetal thrombotic vasculopathy were commonly found placental lesions in NE patients (*n* = 23) (27). Placental lesions indicating thrombosis and reduced fetoplacental flow are significant and independent risk factors for NE (*n* = 93) (28). The presence of more than one placental lesion increases the odds of NE and but there was no correlation between placental pathology and pattern of injury on MRI (*n* = 56) (29). Abnormal placental pathology was found in 29% of neonates with sentinel events and 73% of infants without sentinel events (*p* = 0.0001) who underwent therapeutic hypothermia (30). Placental inflammatory lesions were more commonly seen in neonates without sentinel events (*p* = 0.002) (30). Chronic villitis was associated with basal ganglia injury (BGT) whereas presence of nucleated red blood cells, decreased placental maturation and chronic villitis also resulted in white matter/watershed injury along with BGT involvement (*n* = 95) (31).

The evidence linking chorioamnionitis with NE is variable. It is hypothesized that the timing of infection and degree of inflammatory response can result in either preconditioning or sensitization and can cause either a protective effect or further enhancement of perinatal brain injury, respectively (32). This effect has been shown in animal models. Lipopolysaccharide (LPS) administered 4–6 h prior to 20 and 50 min HI exposure resulted in increased brain injury. However, administration of LPS 24 h prior to 50 min HI exposure resulted in significant attenuation of brain injury (32). CA was found in 8 neonates with signs of perinatal asphyxia (total *n* = 23) (27). Infants with NE and TH had higher odds of having CA compared to infants who did not receive TH (*n* = 98) (33). CA, vasculitis and funisitis was found associated with NE grade I, villitis with NE II and funisitis with grade III in 141 neonates with NE and 309 controls (29). CA was associated with lower risk of brain injury and poor cognitive outcome when compared to non-CA (adjusted OR 0.3; 95% CI 0.1–0.7, *p* = 0.004) in neonates with NE (*n* = 258) although only 20 neonates had histological evidence of CA. In the same cohort neonates with sign of sepsis had higher chances of watershed area injury and abnormal neuromotor scores than those without any clinical signs (*p* = 0.007) (34). In 120 infants with a combination of perinatal acidosis, NE and chorioamnionitis with or without fetal response and patchy/diffuse chronic villitis were independently associated with severity of NE (35). The only individual predictor of abnormal neurodevelopmental outcome at 22–24 months following hypothermia therapy was diffuse chronic villitis. Histological CA had a poor predictive value for development of encephalopathy and death in NE (*n* = 51) (36) but maternal fever (*n* = 336) in labor was an independent factor for NE (adjusted OR 4.72, 95% CI 1.28–17.4) (37). Clinical chorioamnionitis was associated with higher cord blood levels of IL-6, IL-8 and regulated on activation normal T-cell expressed and secreted (RANTES) in NE (*n* = 61). In a cohort of 67 neonates with NE chorioamnionitis whether clinical or histological was associated with persistent metabolic acidosis and may predict poor neurological outcome (38).

Placental pathology has been described as the “blackbox” of pregnancy. Standardized classification of placental pathology findings are essential to further delineate etiology in NE (39).

## Metabolic Disorders

Metabolic disorders are rare causes of NE but should always be considered. Inborn errors of metabolism (IEM) present in the neonatal period after a normal interval of being well and the lack of signs of perinatal asphyxia, but neurologic and multiorgan involvement can present as NE (40). Mitochondrial disorders are also an important cause of NE (41).

Amplitude integrated electroencephalogram (aEEG) was abnormal in 27 neonates with IEM, with the most consistent feature being abnormal background activity and seizures. There was no difference in the aEEG for encephalopathy associated with IEM or NE and differentiation could only be made on clinical suspicion (42). The only metabolic disorder with a diagnostic EEG is non-ketotic hyperglycinemia with an initial “suppression-burst” pattern, changing to hypsarrhythmia during early or mid-infancy and is diagnosed by measuring cerebrospinal fluid glycine levels (42). There are also individual case reports of IEM such as sulfite oxidase and cytochrome C deficiency presenting as NE (43, 44).

MRI findings can vary depending on the type of inborn error of metabolism (IEM) and can vary from white matter lesions to deep gray nuclei involvement. Mitochondrial encephalopathies have a symmetrical bilateral distribution and can be diagnosed as NE initially (45). Metabolic disorders should be considered if there is no clear intrapartum event, persistent lactic acidosis and/or hypoglycemia (45) and workup commenced. Different patterns of injury on MRI brain might aid in differentiating metabolic disorders from NE. Globus Pallidus involvement is common in IEM compared to NE although pattern of injury in sulfite oxidase deficiency and molybdenum factor deficiency can mimic NE (46).

Isolated seizures are associated with disorders such as: pyridoxine-responsive seizures, non-ketotic hyperglycinemia (NKH), dihydropyrimidine dehydrogenase deficiency, sulfite oxidase deficiency (either isolated or as part of molybdenum cofactor deficiency), 4-aminobutyrate aminotransferase (GABA transaminase) deficiency, folinic acid-responsive seizures, 3-phosphoglycerate dehydrogenase deficiency, guanidinoacetate methyltransferase (GAMT) deficiency, and glucose transporter (GLUT-1) deficiency. Babies with multiorgan involvement, seizures, metabolic acidosis, lactic academia, hyperammonemia may have: organic acidaemias, urea cycle defects, peroxisomal disorders (e.g., Zellwegers syndrome) or mitochondrial disorders.

A detailed family history to establish risk factors such as parental consanguinity or a previously affected child are important as most inborn errors of metabolism have autosomal recessive or maternal inheritance. Baseline investigations such as electrolytes, ammonia, serum amino acid, lactate, acylcarnitine and urine organic acid may be considered in NE (40). There are challenges to metabolic testing as they may need to be repeated if the baby is very unwell with multiorgan dysfunction at the time of sampling (46). Recent research has shown great potential for metabolomics as biomarkers of NE. Succinate has been shown to be elevated in children with NE who were severely affected (47).

## Thrombophilia and Neonatal Stroke

Asphyxia (in 4% of cases) has been implicated in pathogenesis of neonatal stroke (48). Focal clonic seizures are the most common presentation of neonatal stroke (49) and 6 infants with stroke were found in a cohort of NE (*n* = 124) (50). These babies typically presented with seizures and had worse neurodevelopmental outcome than the rest of the cohort. Blood clotting disorders such as polycythemia, factor V Leiden, hemoglobin disorders, and prothrombin mutations were not found in 4 of the 6 patients that were investigated (50).

In animal models increased homocysteine levels resulted in an increase in proinflammatory cytokines such as tumor necrosis factor alpha (TNFα), interleukin 1 beta (IL-1β) and IL-6 in hippocampus and cerebral cortex. This proinflammatory state might be responsible for neuronal and cerebrovascular effects seen in adults (51). The role of homocysteine or polymorphisms in methylenetetrahydrofolate reductase gene in causation of neonatal brain injury is not clear (52). In a cohort of 118 neonates with NE, white matter/watershed injury was found to be more likely to be associated with hypoglycemia, polymorphism in methylenetetrahydrofolate reductase (MTHFR) and plasma homocysteine levels in the upper quartile of normal (52).

## Genetic and Epigenetic Abnormalities

There is lack of published data linking genetic predisposition to development of NE. In 40 patients with NE, the genetic variation in the prothrombic factors such as prothrombin G20210A, factor V Leiden G1691A and methylenetetrahydrofolate reductase [MTHFR] C677T and Tumor necrosis factor gene had no effect on NE grading, EEG, death, cranial ultrasound (US), neurologic outcome at discharge, 6 and 12 months. Polymorphism in interleukin-6 gene (IL-6 174GC) had a protective effect and was associated with normal EEG, cranial US and neurologic examination at discharge (53). In a retrospective case control study of 11 neonates with NE, mothers were found to have higher incidence of MTHFR polymorphism (7 homozygous and 4 heterozygous) when compared to controls (54). Genetic polymorphism may increase the risk or severity of neonatal brain injury in NE and should be considered (53).

The Avon Longitudinal Study of Parents and Children with a total of 7,611 term infants were genotyped and exposure measures were the presence of one or more minor alleles in one of 3 SNPs (rs2284411, rs2498804, rs1835740). The primary outcome was the need for resuscitation at birth, which was associated with rs1835740 (55). Larger international collaborative studies are required to develop the required patient numbers to explore genetic connections and to subdivide patients by etiology.

Another category of babies with a genetic cause for neonatal encephalopathy are those with neonatal myopathies and encephalopathies. Patients who have inherited myopathies (centronuclear myopathy) may have neonatal distress, hypotonia, and signs suggestive of HIE (56). Congenital myotonic dystrophy type 1 is characterized by hypotonia and severe generalized weakness at birth, often with respiratory insufficiency, intellectual disability and early death. It is caused by expansion of a CTG trinucleotide repeat in the non-coding region of *DMPK*.

In the future detailed genotyping including easier access to microarray techniques in the clinical setting will provide more information to allow individualized care and potentially classify infants by genotype.

## Neuroimaging

Different patterns of injury can be seen on MRI but this does not out rule HIE as the cause of injury. The pattern of injury depends on the severity, duration and repetitiveness of the hypoxic ischemia and can lead to involvement of basal ganglia, thalami, brain stem and/ or cerebral white matter in different combinations (1). With profound hypoxia the predominant injury is in deeper gray structures that is basal ganglia and thalami. This is explained by the fact that these areas are myelinated and have higher metabolic rate. Conditions that reduced rather than cause complete cessation of blood flow such as anemia and hypovolemia are associated with injury in cerebral cortex and deeper gray structures are preserved (57). In a cohort of 245 patients with NE 197 (80%) had MRI findings consistent with acute perinatal insult. MRI in 40 infants (16%) were normal. 9 infants (4%) had shown sign of thrombosis/infarction, genetic disorders, antenatal insult and mitochondrial (complex I deficiency) on MRI in addition to hypoxic ischemic insult. Neuroimaging in 8(3%) infants with clinical diagnosis of NE showed either inborn error of metabolism, neuromuscular disorder or no diagnosis and these findings were not consistent with hypoxic ischemia (48). Placental high grade villitis of unknown etiology (*n* = 4) was found in a cohort of 36 patients associated with white matter injury secondary to inflammation/ oxidative stress in perinatal period (58).

## Implications for Therapy

All infants with NE might not benefit equally from hypothermia. Therefore, detecting the accurate etiology in each case of NE has implications for treatment as there are some treatable causes, which need rapid definition for optimal management. The use of bacterial PCR for the detection of infection in neonates with NE may be more beneficial than blood culture alone with a low bacterial yield and also hypothermia may decrease the inflammatory response. Placental histology reflects intrauterine environment and might clarify the underlying etiology. All neonates at risk of IEM should be investigated for metabolic disorders as these patients might benefit from specific therapies rather than hypothermia. Similarly thrombophilia screen should be considered in at risk patients such as patients with stroke, family history of thrombophilia. Detailed evaluation of the etiology of NE is important to identify treatable causes and categorize this heterogenous group of neonates so that appropriate treatment can be tailored according to the etiology and improvement in longterm outcome. Similarly it will be useful to stratify large multicenter cohorts of infants with NE by etiology to fully understand the etiology, the response to hypothermia treatment and longterm outcome.

## Author Contributions

SA: primary author; TS: helped with search for relevant articles and figure; EM: supervising author.

### Conflict of Interest Statement

The authors declare that the research was conducted in the absence of any commercial or financial relationships that could be construed as a potential conflict of interest.
